# Factors Associated With Mosaicism in Human Embryos: A Retrospective Study

**DOI:** 10.7759/cureus.62967

**Published:** 2024-06-23

**Authors:** Long Nu-Hai Nguyen, Huy Phuong Tran, Vy Nguyen-Thao Do, Loc Thai Ly, Tuyet Thi-Diem Hoang

**Affiliations:** 1 Infertility Department, Hung Vuong Hospital, Ho Chi Minh City, VNM; 2 Medical Genetics Department, Hung Vuong Hospital, Ho Chi Minh City, VNM

**Keywords:** human embryo, biopsy, mosaicism, preimplantation genetic testing, ivf

## Abstract

Objective

This study aims to identify factors associated with mosaicism in human embryos at Hung Vuong Hospital.

Methods

We performed a retrospective analysis of data from 2018 to 2022, approved by the Hung Vuong Hospital Ethics Committee (CS/HV/23/15). We analyzed variables such as demographic characteristics, clinical measurements, and in-vitro fertilization (IVF) cycle outcomes to investigate their relationship with embryo mosaicism.

Results

A total of 73 couples undergoing IVF with preimplantation genetic testing (PGT) were included in the analysis. Among 308 embryos, 98 (31.8%) were mosaic, 124 (40.3%) were euploid, and 86 (27.9%) were aneuploid. Univariable analysis revealed that female age was significantly associated with increased odds of mosaicism (odd ratio (OR) = 1.11, 95% confidence interval (CI): 1.04 - 1.19, p = 0.003). Male age demonstrated a marginal association with mosaicism (OR = 1.05, 95% CI: 1.00 - 1.11, p = 0.07). Other factors, including body mass index (BMI), anti-Mullerian hormone (AMH) levels, blood types, and sperm quality, were not significantly associated with mosaicism. In the multivariable analysis, controlling for both female and male age, female age showed a trend toward significance (OR = 1.12, 95% CI: 1.02 - 1.23, p = 0.02), while male age showed no significant effect (OR = 0.99, 95% CI: 0.92 - 1.06, p = 0.75).

Conclusions

The findings suggest that female age is a critical factor influencing the occurrence of mosaicism in embryos. Further research is needed to fully understand the mechanisms underlying mosaicism in human embryos.

## Introduction

After decades of development, in vitro fertilization (IVF) has revolutionized reproductive medicine by allowing couples with infertility to achieve pregnancy through the fertilization of oocytes with sperm outside the body, followed by the transfer of cultured embryos into the uterus [1]. In the era of information technology and the post-COVID-19 world, telemedicine is increasingly being incorporated into IVF practices to offer enhanced patient support [2]. As technology has advanced, several complementary techniques have emerged, enhancing the efficacy and safety of IVF. One such advancement is cryopreservation of embryos, which involves the freezing of embryos at various stages of development and has become a cornerstone of modern IVF practices [3]. This process allows for the storage of embryos, provides flexibility in timing for embryo transfer, and enables multiple attempts without the need for repeated ovarian stimulation. Consequently, frozen embryo transfer (FET) has emerged as a significant trend, where cryopreserved embryos are transferred in subsequent cycles, reducing the risk of ovarian hyperstimulation syndrome [4]. 

Preimplantation genetic testing (PGT) is a valuable tool for the genetic screening of embryos before transfer. This test can identify chromosomal abnormalities such as aneuploidies and structural rearrangements, thereby increasing the chance of successful implantation and reducing the risks of miscarriage and genetic disorders. Despite its advantages, PGT has uncovered a phenomenon known as mosaicism. Mosaic embryos, which contain both normal and abnormal cells, are associated with unfavorable outcomes in IVF, including reduced implantation rates, increased miscarriage rates, and lower live birth rates [5]. While some mosaic embryos can result in healthy live births [6], others may cause genetic disorders or developmental delays in offspring [7]. Therefore, it is crucial for clinicians and patients to carefully consider the use of mosaic embryos in fertility treatments due to their potential negative impact on the IVF success rate.

Although the exact causes of mosaicism in human embryos remain unclear, several potential contributing factors have been identified. Maternal age is a significant risk factor, and its association with aneuploid embryos is well documented [8]. Its correlation with mosaicism has also been confirmed in a meta-analysis [9]. Additionally, paternal age or male factors have been linked to mosaicism in embryos [10]. Poor-quality embryos or those developing slowly are more likely to exhibit mosaicism [11]. Furthermore, studies have shown associations between mosaicism and semen quality, fertilization methods, and biopsy protocols [12,13].

In summary, there is still considerable debate about the factors associated with mosaicism, and further research is needed to fully understand the mechanisms underlying this phenomenon. This study aims to investigate the determinants of mosaicism in human embryos, focusing on patient demographics, embryo characteristics, and clinical procedures. By exploring these aspects, the study seeks to contribute valuable insights to the ongoing debate on this topic.

## Materials and methods

Study design and population 

This retrospective analysis includes all couples who attempted to conceive using IVF with PGT at the Infertility Department of Hung Vuong Hospital from December 2018 to December 2022. The study was approved by the Hung Vuong Hospital Ethics Committee (CS/HV/23/15). Couples were eligible if they had biopsied embryos that underwent PGT and had complete data on embryo ploidy status. Exclusion criteria included couples with no available blastocysts for biopsy and biopsied embryos that either failed amplification or underwent amplification without genetic testing. 

Patient and embryo data were collected from medical records. Baseline characteristics of the patients included female and male age, body mass index (BMI), anti-Mullerian hormone (AMH) levels, blood types, type of infertility, indications for PGT, karyotype results, sperm quality, and oocyte parameters. Embryo characteristics included embryo quality, biopsy day, and ploidy status (euploidy, aneuploidy, or mosaicism). Embryo quality was assessed based on morphological criteria [14]. Biopsies were performed on embryos on day four or day five of development.

Assisted reproductive technologies (ART) cycle and laboratory procedures

Ovarian stimulation was performed in accordance with published standard guidelines. Oocytes were collected 36 hours after ovulation induction. All embryos were continuously cultured in Continuous Single Culture-NX Complete (CSCM-NXC, Fujifilm, Irvine Scientific, USA) microdroplets until reaching the blastocyst stage. Following a biopsy, each biopsied embryo was independently vitrified. Trophectoderm biopsies were then analyzed using next-generation sequencing (NGS).

Statistical analysis

Statistical analyses were conducted using generalized estimating equations (GEE). The initial step involved a univariable analysis to examine the relationship between each baseline characteristic and the likelihood of mosaicism, calculating the odd ratio (OR) with a 95% confidence interval (CI) and corresponding p-values for each factor. Characteristics that showed significant associations in the univariable analysis were subsequently included in a multivariable GEE model to identify independent predictors of mosaicism. The threshold for statistical significance was set at p < 0.05. All analyses were performed using R software.

## Results

Table 1 outlines the baseline characteristics of the 73 patients involved in the study. Among the female patients, the mean age is 35.52 years, with a standard deviation of 4.72 years. Conversely, male patients exhibit a slightly higher mean age of 38.86 years, with a standard deviation of 5.89 years. The average BMI of the patients is 21.41 kg/m², with a standard deviation of 2.22 kg/m², indicating that most individuals fall within the normal BMI range. Additionally, AMH levels, which provide an indication of ovarian reserve, average at 3.70 ng/mL with a standard deviation of 2.57. Among both female and male patients, blood type O is the most prevalent, found in 26 females (35.6%) and 29 males (39.7%).

**Table 1:** Baseline characteristics of the included patients SD: standard deviation; PGT: preimplantation genetic testing

Characteristics	Total (n=73)
Female age, years (mean ± SD)	35.52 ± 4.72
Male age, years (mean ± SD)	38.86 ± 5.89
Body mass index, kg/m^2^ (mean ± SD)	21.41 ± 2.22
Anti-Mullerian hormone, ng/mL (mean ± SD)	3.70 ± 2.57
Female blood type, n (%):	
1/ A	19 (26)
2/ B	19 (26)
3/ O	26 (35.6)
4/ AB	9 (12.4)
Male blood type, n (%):	
1/ A	13 (17.8)
2/ B	24 (32.9)
3/ O	29 (39.7)
4/ AB	7 (9.6)
Type of infertility, n (%)	
1/ Primary infertility	20 (27.4)
2/ Secondary infertility	53 (72.6)
Indication for PGT, n (%):	
1/ Elderly couple	9 (12.3)
2/ Repeated implantation failure	3 (4.1)
3/ Repeated pregnancy loss	21 (28.8)
4/ Abnormal genetics	40 (54.8)
Female karyotype result, n (%):	
1/ Normal	63 (86.3)
2/ Abnormal	10 (13.7)
Male karyotype result, n (%):	
1/ Normal	58 (79.5)
2/ Abnormal	15 (20.5)
Sperm quality, n (%):	
1/ Normal	49 (67.1)
2/ Abnormal	21 (28.8)
3/ Surgical	3 (4.1)
No. of retrieved oocytes (mean ± SD)	16.48 ± 7.25
No. of mature oocytes (mean ± SD)	13.00 ± 6.11
No. of immature oocytes (mean ± SD)	2.21 ± 2.31
No. of abnormal oocytes (mean ± SD)	1.07 ± 1.68

In terms of reproductive status, secondary infertility is more common, affecting 53 patients (72.6%), compared to primary infertility, which affects 20 patients (27.4%). Most patients exhibit normal karyotypes, with 58 males (79.5%) and 63 females (86.3%). Sperm quality is classified as normal in 49 cases (67.1%), while 21 cases (28.8%) show abnormal sperm parameters. Regarding the outcomes of the ovum pick-up process, patients had an average retrieval of 16.48 oocytes per cycle, with an average of 13.00 being mature. The mean counts of immature and abnormal oocytes were 2.21 and 1.07, respectively.

Table 2 presents the baseline characteristics of the 308 embryos analyzed in the study. The majority of embryos, 270 (87.7%), were classified as having good quality, while 38 (12.3%) were categorized as not good. Most biopsies, 254 (82.5%), were performed on day five, compared to 54 (17.5%) on day four. Regarding ploidy status, 124 embryos (40.3%) were euploid, 86 (27.9%) were aneuploid, and 98 (31.8%) were mosaic. 

**Table 2:** Baseline characteristics of the included embryos

Characteristics	Total (n = 308)
Embryo quality, n (%):	
1/ Good	270 (87.7)
2/ Not good	38 (12.3)
Biopsy day, n (%):	
1/ Day 4	54 (17.5)
2/ Day 5	254 (82.5)
Ploidy status, n (%):	
1/ Euploidy	124 (40.3)
2/ Aneuploidy	86 (27.9)
3/ Mosaic	98 (31.8)

Table 3 provides valuable insights into the factors associated with mosaicism in human embryos. The results indicate that female age is significantly associated with an increased risk of mosaicism, with an OR of 1.11 (95% CI: 1.04 - 1.19, p = 0.003). Male age, with an OR of 1.05 (95% CI: 1.00 - 1.11, p = 0.07), indicating a potential, albeit weaker, association with mosaicism. However, BMI and AMH levels do not show significant associations with mosaicism, with p-values of 0.33 and 0.51, respectively. This suggests that factors related to body weight and ovarian reserve may not be major determinants of mosaicism in embryos. Both female and male blood types also do not demonstrate significant impacts on mosaicism risk. Indications for PGT do not show significant associations with type of infertility, karyotype results for both females and males, as well as sperm quality and various parameters related to oocyte retrieval, do not exhibit significant associations with mosaicism, suggesting that these factors may not have an influence on the occurrence of mosaic embryos. Similarly, embryo quality and biopsy day also do not demonstrate significant associations with mosaicism.

**Table 3:** Univariable analysis using generalized estimating equations (GEE) PGT: preimplantation genetic testing; OR: odds ratio; CI: confidence interval A p-value of <0.05 was considered significant.

Characteristics	OR	95% CI	P-value
Female age	1.11	[1.04 - 1.19]	0.003
Male age	1.05	[1.00 - 1.11]	0.07
Body mass index	1.06	[0.94 - 1.21]	0.33
Anti-Mullerian hormone	0.96	[0.86 - 1.08]	0.51
Female blood type:			
1/ A	Reference		
2/ B	0.93	[0.46 - 1.86]	0.83
3/ O	1.11	[0.56 - 2.22]	0.76
4/ AB	1.92	[0.74 - 4.97]	0.18
Male blood type:			
1/ A	Reference		
2/ B	1.65	[0.72 - 3.76]	0.23
3/ O	1.70	[0.75 - 3.86]	0.21
4/ AB	1.37	[0.47 - 4.00]	0.57
Type of infertility:			
1/ Primary infertility	Reference		
2/ Secondary infertility	0.57	[0.31 - 1.06]	0.08
Indication for PGT:			
1/ Elderly couple	Reference		
2/ Implantation failure	0.60	[0.11 - 3.24]	0.55
3/ Pregnancy loss	0.60	[0.19 - 1.88]	0.38
4/ Abnormal genetics	0.55	[0.18 - 1.70]	0.30
Female karyotype result:			
1/ Normal	Reference		
2/ Abnormal	1.05	[0.49 - 2.25]	0.90
Male karyotype result:			
1/ Normal	Reference		
2/ Abnormal	1.05	[0.56 - 1.97]	0.88
Sperm quality:			
1/ Normal	Reference		
2/ Abnormal	0.99	[0.54 - 1.80]	0.97
3/ Surgical	1.71	[0.37 - 7.91]	0.49
No. of retrieved oocytes	0.98	[0.94 - 1.01]	0.22
No. of mature oocytes	0.99	[0.95 - 1.03]	0.67
No. of immature oocytes	0.91	[0.81 - 1.02]	0.09
No. of abnormal oocytes	0.86	[0.72 - 1.03]	0.11
Embryo quality:			
1/ Good	Reference		
2/ Not good	1.17	[0.47 - 2.87]	0.74
Biopsy day:			
1/ Day 4	Reference		
2/ Day 5	1.13	[0.55 - 2.32]	0.74

Table 4 illustrates the multivariable analysis to evaluate the independent effects of female and male age on the likelihood of mosaicism in embryos. The adjusted OR for female age was 1.12 (95% CI: 1.02 - 1.23, p = 0.02), suggesting a trend where increasing female age might be associated with a significantly higher risk of mosaicism. Conversely, the adjusted OR for male age was 0.99 (95% CI: 0.92 - 1.06, p = 0.75), indicating no significant association between paternal age and the risk of mosaicism.

**Table 4:** Multivariable analysis using generalized estimating equations (GEE) OR: odds ratio; CI: confidence interval A p-value of <0.05 was considered significant.

Characteristics	OR	95% CI	P-value
Female age	1.12	[1.02 - 1.23]	0.02
Male age	0.99	[0.92 - 1.06]	0.75

## Discussion

This study identifies a significant association between maternal age and the risk of mosaicism in human embryos, with our findings demonstrating that increasing female age correlates with a higher likelihood of mosaicism. This aligns with existing research that underscores advanced maternal age as a critical risk factor for chromosomal abnormalities, including mosaicism, likely due to age-related declines in oocyte quality and increased chromosomal segregation errors [8,9]. Although the association between male age and mosaicism was not statistically significant in our multivariable analysis, it exhibited a potential trend that warrants further investigation, especially in light of a study suggesting that advanced paternal age can contribute to mosaicism [10]. Furthermore, male factors such as semen quality have been linked to mosaicism in other studies [12,15]. Our findings did not show significant associations for other factors such as BMI, AMH levels, or blood types. Moreover, embryo quality, while not significantly associated with mosaicism in our study, has been debated in the literature, with a report indicating that poor-quality embryos may increase the incidence of mosaicism [11]. 

The significant association of maternal age with mosaicism aligns with the broader understanding of reproductive aging and its impact on genetic stability within oocytes. As women age, the risk of chromosomal segregation errors increases, leading to a higher incidence of mosaicism in embryos. Indeed, a study has shown that the lowest risk for embryonic aneuploidy occurs between ages 26 and 30, with both younger and older age groups exhibiting higher rates of aneuploidy and a greater risk of complex aneuploidies [16]. This underscores the importance of considering maternal age in clinical decision-making and counseling for couples undergoing IVF and PGT. In contrast, paternal age does not appear to be a major determinant of mosaicism, suggesting that factors related to maternal characteristics may play more pivotal roles in the development of mosaicism.

Our study has several limitations. Firstly, its retrospective design may introduce biases related to data collection. Secondly, the nature of embryo datasets can result in the same data being repeated due to one couple often having several embryos in one IVF cycle. Thirdly, being conducted at a single center, Hung Vuong Hospital, the study's findings may not be applicable to other settings or populations. Additionally, the study examined a limited set of factors associated with mosaicism, leaving out other potential contributors such as patient lifestyles, environmental exposures, and detailed genetic backgrounds. Furthermore, the available scientific evidence regarding the causes of embryonic mosaicism is limited, with different studies reaching varying conclusions, leading to considerable debate. The incidence of mosaic embryos may vary by the nature of each IVF center, and a study has concluded that biopsy technicians may contribute to artificial mosaicism as an extrinsic factor [12]. Additionally, other studies have suggested that the detection system and biopsy protocol also influence the incidence of mosaicism [12,13]. These factors limit the comprehensiveness of our analysis and understanding of all possible factors influencing mosaicism. 

Despite its limitations, this study has important implications for reproductive medicine. The significant association of maternal age with the risk of mosaicism underscores the need for personalized reproductive counseling. Patients can be better informed about the potential risks related to advanced maternal age and mosaicism before undergoing an IVF cycle with PGT. Given the diverse factors that can influence mosaicism and the ongoing debate surrounding them, further research, particularly multi-center studies, is crucial.

## Conclusions

Our study suggests that female age is a critical factor influencing the occurrence of mosaicism in embryos. This finding aligns with existing literature that associates increased maternal age with higher risks of chromosomal segregation errors, leading to mosaicism. While male age did not show a significant effect in our multivariable analysis, its potential association with mosaicism suggests that further investigation is warranted. Other factors, such as BMI, AMH levels, and blood types, were not found to significantly influence mosaicism in our cohort.

These insights emphasize the need for age-specific counseling in IVF cycles with PGT. Furthermore, this study highlights the importance of continuous research into the mechanisms of mosaicism, including factors beyond those examined in our analysis. Future studies, particularly those with multi-center designs, are essential to explore additional contributors to mosaicism.
